# Supplementary material for: HIVprotI: an integrated web based platform for prediction and design of HIV proteins inhibitors
Source: J Cheminform. 2018 Mar 9;10:12. doi: 10.1186/s13321-018-0266-y (PMC5845081; doi:10.1186/s13321-018-0266-y)
Supplement: Supplementary file 2 — Additional file 2. Source code of HIVProtI web server. [file 13321_2018_266_MOESM2_ESM.zip › HIVprotI_Source-code/hivprotI/weka-3-6-6/doc/weka/gui/Loader.html]

Loader


---


|  |  |  |
| --- | --- | --- |
| **Overview** \| **Package** \| **Class** \| **Tree** \| **Deprecated** \| **Index** \| **Help** \| **Weka's home** | |  |
| **PREV CLASS**   **NEXT CLASS** | **FRAMES**    **NO FRAMES**     **All Classes** |  |
| SUMMARY: NESTED \| FIELD \| CONSTR \| METHOD | DETAIL: FIELD \| CONSTR \| METHOD |  |


---


## weka.gui Class Loader

```
java.lang.Object
  weka.gui.Loader
```

---

``` public class Loader extends java.lang.Object ```

This class is for loading resources from a JAR archive.

**Version:**
:   $Revision: 1.2 $

**Author:**
:   FracPete (fracpete at waikato dot ac dot nz)

---

**Constructor Summary**

`Loader(java.lang.String dir)`
  
          initializes the object


| **Method Summary** | |
| --- | --- |
| `java.lang.String` | `getDir()`             returns the dir prefix |
| `java.io.InputStream` | `getInputStream(java.lang.String filename)`             returns an InputStream for the given filename, can be NULL if it fails |
| `static java.io.InputStream` | `getInputStream(java.lang.String dir, java.lang.String filename)`             returns an InputStream for the given dir and filename, can be NULL if it fails |
| `java.io.Reader` | `getReader(java.lang.String filename)`             returns a Reader for the given filename, can be NULL if it fails |
| `static java.io.Reader` | `getReader(java.lang.String dir, java.lang.String filename)`             returns a Reader for the given filename and dir, can be NULL if it fails |
| `java.net.URL` | `getURL(java.lang.String filename)`             returns a URL for the given filename, can be NULL if it fails |
| `static java.net.URL` | `getURL(java.lang.String dir, java.lang.String filename)`             returns a URL for the given filename, can be NULL if it fails |
| `java.lang.String` | `processFilename(java.lang.String filename)`             returns the processed filename, i.e. |

**Methods inherited from class java.lang.Object**

`equals, getClass, hashCode, notify, notifyAll, toString, wait, wait, wait`

**Constructor Detail**

### Loader

```
public Loader(java.lang.String dir)
```

:   initializes the object


**Method Detail**

### getDir

```
public java.lang.String getDir()
```

:   returns the dir prefix

---


### processFilename

```
public java.lang.String processFilename(java.lang.String filename)
```

:   returns the processed filename, i.e. with the dir-prefix if it's
    missing

---


### getURL

```
public static java.net.URL getURL(java.lang.String dir,
                                  java.lang.String filename)
```

:   returns a URL for the given filename, can be NULL if it fails

---


### getURL

```
public java.net.URL getURL(java.lang.String filename)
```

:   returns a URL for the given filename, can be NULL if it fails

---


### getInputStream

```
public static java.io.InputStream getInputStream(java.lang.String dir,
                                                 java.lang.String filename)
```

:   returns an InputStream for the given dir and filename, can be NULL if it
    fails

---


### getInputStream

```
public java.io.InputStream getInputStream(java.lang.String filename)
```

:   returns an InputStream for the given filename, can be NULL if it fails

---


### getReader

```
public static java.io.Reader getReader(java.lang.String dir,
                                       java.lang.String filename)
```

:   returns a Reader for the given filename and dir, can be NULL if it fails

---


### getReader

```
public java.io.Reader getReader(java.lang.String filename)
```

:   returns a Reader for the given filename, can be NULL if it fails


---


|  |  |  |
| --- | --- | --- |
| **Overview** \| **Package** \| **Class** \| **Tree** \| **Deprecated** \| **Index** \| **Help** \| **Weka's home** | |  |
| **PREV CLASS**   **NEXT CLASS** | **FRAMES**    **NO FRAMES**     **All Classes** |  |
| SUMMARY: NESTED \| FIELD \| CONSTR \| METHOD | DETAIL: FIELD \| CONSTR \| METHOD |  |


---
